# La comunicación como herramienta estratégica de la atención centrada en la persona

**DOI:** 10.23938/ASSN.1106

**Published:** 2024-12-27

**Authors:** María Sánchez-Marco, Antonio Esteve-Ríos, Silvia Escribano

**Affiliations:** 1 Universidad de Alicante Facultad de Ciencias de la Salud Departamento de Enfermería San Vicente del Raspeig Alicante España; 2 Instituto de Investigación Sanitaria y Biomédica de Alicante (ISABIAL) Alicante España

El Modelo de Atención Centrada en la Persona se ha convertido en una prioridad en la política sanitaria mundial, reconociendo su importancia como componente fundamental de la calidad de la atención sanitaria^1^. Desarrollado por McCormack y McCance^2^ en 2006 y adaptado al contexto español en 2022 por Chopenera y col^3^, este enfoque sitúa a las personas en el centro de la atención de los sistemas sanitarios, respetando sus preferencias y expectativas, y considerándolas agentes clave en la toma de decisiones sobre su propia salud^3^. A pesar de que pueden encontrarse diferentes definiciones del término, la literatura identifica aspectos centrales inherentes a este enfoque de atención^4^: 1) Empatía, compasión, apoyo emocional y comprensión; 2) Respeto a las creencias, los valores y la dignidad; 3) Compromiso como presencia; 4) Relación en la atención: asociación, confianza mutua y relación terapéutica; 5) Comunicación e intercambio de la información; 6) Toma de decisiones compartida: empoderamiento, autonomía y participación en el tratamiento; 7) Enfoque holístico: perspectiva biopsicosocial, se consideran relevantes los problemas no médicos y el impacto del contexto; 8) Enfoque individualizado: considerar aspectos específicos de la vida y preferencias y 9) Atención coordinada: coordinación en todo el sistema de salud, colaboración interprofesional y coordinación a lo largo del tiempo.

Instituciones internacionales como la Organización Mundial para la Salud (OMS) en diversos informes^5^, el Consejo Internacional de Enfermeras^6^ o el Instituto *Picker Europe*^7^ han establecido estrategias globales para impulsar y establecer este paradigma de atención en todos los sistemas sanitarios para que sean capaz de adaptarse a los nuevos retos. El envejecimiento de la población ligado al aumento de la prevalencia de las afecciones crónicas de larga duración es un ejemplo de estos retos, que requerirá una prestación de servicios compleja, continua, segura, sostenible y que parta de las necesidades de las personas, familias y comunidades^8^^,^^9^. En este contexto la curación dejará de ser el objetivo principal, cobrando especial relevancia la atención de la persona desde una perspectiva integral e interdisciplinar^10^.

Para ello, es prioritario que los profesionales cuenten con un nivel de competencia adecuado que les capacite en dicho paradigma de atención; destacando las habilidades de comunicación y las relaciones interpersonales como algunos de los elementos clave para su adecuada implementación^11^. La adquisición de habilidades de comunicación en los profesionales sanitarios se ha convertido en una cuestión central a nivel internacional y en el ámbito europeo^12^. La comunicación clínica efectiva se relaciona con resultados positivos en la calidad de la atención y con una mejor salud física y emocional de la persona^13^^,^^14^ y con mejores habilidades de autocuidado, autonomía, bienestar y satisfacción con la experiencia de la atención^5^^,^^15^. Sin embargo, sigue siendo necesario desarrollar dichas habilidades. La literatura muestra un déficit en las habilidades de comunicación de los profesionales sanitarios^16^^,^^17^, incluso en contextos sanitarios complejos donde aumenta la necesidad de ser competente en dichas habilidades, como son los cuidados paliativos y al final de la vida^18^. Además, se encuentran resultados ambiguos respecto a la percepción por los propios profesionales sanitarios sobre sus habilidades de comunicación, desde una percepción deficiente^19^ a una sobreestimada^20^, en la que los profesionales evalúan más satisfactoriamente su competencia comunicativa que las personas que reciben la atención.

La implementación del Modelo Centrado en la Persona también encuentra dificultades en relación a la falta de promoción e impulso por parte de los sistemas sanitarios^21^ y de las instituciones educativas que forman a los profesionales^22^^,^^23^. Estas instituciones mantienen un enfoque predominantemente biomédico y centrado en la patología, en lugar de orientarse hacia las necesidades integrales de la persona^24^^,^^25^. En consecuencia, la implementación de modelos no centrados en la persona tiene un enfoque excesivamente especializado y tecnificado de los profesionales sanitarios, enfocado en la búsqueda e identificación del diagnóstico y en la aplicación de tratamientos, perdiendo la visión de la persona como una entidad compleja que se desarrolla en un contexto determinado, y deshumanizando la atención sanitaria ofrecida^25^. Esta situación se ve agravada por la falta de recursos humanos y la mayor sobrecarga asistencial que limita el tiempo dedicado a la atención, situando la comunicación clínica en un segundo plano^19^^,^^26^.

Para poder superar estas barreras, es fundamental implementar un enfoque sistemático en todos los niveles de la organización. Es evidente la necesidad de fortalecer la conexión entre la evidencia y la práctica clínica^27^, además de sensibilizar a todas las instituciones implicadas, fomentando su compromiso para la integración de una cultura centrada en el Modelo de Atención Centrado en la Persona como prioridad estratégica de sus agendas sanitarias. No obstante, el cambio de cultura y la mejora de los procesos requiere el conocimiento detallado del grado de implementación de este modelo en las instituciones. Esto permitirá identificar los déficits y, a partir de ello, establecer procesos de mejora eficaces y sostenibles. En este sentido, es importante destacar la adaptación y validación de instrumentos que permiten evaluar la práctica centrada en la persona en el ámbito español desde diferentes perspectivas de los agentes implicados, como son las adaptaciones de los instrumentos denominados Person*-Centred Practice Framework (PCPF)* desde la perspectiva de la persona, publicado por Mónica Váquez-Calatayud y col en este número 47(3) de la revista Anales del Sistema Sanitario de Navarra^28^, y *Person-Centred Practice Inventory-Staff (PCPI-S)* desde la perspectiva de los profesionales de la salud^29^. Disponer de herramientas validadas permite diagnosticar la situación actual y, en consecuencia, facilitar la implementación de mejoras eficaces en la atención centrada en la persona dentro del sistema sanitario evaluado^28^^,^^29^. Por último, no deberíamos olvidar, como línea estratégica relacionada directamente con la mejora de los procesos y las buenas prácticas, la necesidad de capacitar a los profesionales acorde con los principios y componentes del Modelo de Atención Centrada en la Persona.

Por todo ello, podemos concluir que sería conveniente: 1) Promover la sensibilización de las instituciones involucradas en el ámbito de la salud y la educación, fomentando su compromiso para integrar la cultura centrada en la persona como prioridad estratégica; 2) Llevar a cabo una evaluación del grado de implementación del Modelo Centrado en la Persona para poder identificar los déficits y establecer mejoras eficaces y sostenibles que influyan en políticas sanitarias; y, 3) Impulsar la capacitación de estudiantes y a los profesionales sanitarios en los principios y componentes del Modelo de Atención Centrada en la Persona, como línea estratégica para mejorar los procesos y las buenas prácticas.
